# 5-Azacytidine in Chronic Myelomonocytic Leukemia: Case Report and Review of Literature.

**DOI:** 10.4084/MJHID.2011.011

**Published:** 2011-03-15

**Authors:** M. Greco, M. Criscuolo, L. Fianchi, E. Fabiani, L. Pagano, MT. Voso

**Affiliations:** Istituto di Ematologia, Università Cattolica Sacro Cuore, Rome, Italy

## Abstract

Hypomethylating drugs are useful in the management of Myelodysplastic syndromes, but there are only few reports on chronic myelomonocycitic (CMML) leukemia patients. We describe our experience in 3 CMML patients treated with azacitidine. Two patients obtained partial response after 4 treatment cycles with only minor toxicity and are in continuous partial response, with stable peripheral blood counts, at 29 and 30 cycles from treatment start.

## Introduction:

Chronic myelomonocytic leukemia (CMML) is a clonal disorder of hematopoietic stem cells often occurring in elderly patients. It was originally classified by the French-American-British (FAB) working group as a myelodysplastic syndrome characterized by monocytosis (> 1000/microl), bone marrow monocyte infiltration, blast cells less than 5% in the peripheral blood and less than 30% in the bone marrow.^1^ In the new WHO classification system of tumors of hematopoietic and lymphoid tissues, CMML has been reclassified as a myelodysplastic/myeloproliferative diseases, a hybrid disorder characterized by proliferation of the myeloid series and dysplasia of erythroid-megakaryocytic series. CMML has been subdivided in the WHO classification in two subclasses according to prognosis, blood and bone marrow blast count: CMML-1: <5% blasts and 5–9% blasts in peripheral blood and bone marrow, respectively, and CMML-2: <10% blasts and 10–19% blasts in peripheral blood and bone marrow, respectively.^2^

CMML is a very hard disease to treat and the prognosis is quite variable, with a median survival of about 20 months. Patients elderly age and comorbidites impact on survival, making bone marrow transplantation only rarely possible. Patients are usually treated with supportive care or mild cytoreductive therapy, like hydroxyurea or low dose cytarabine, until leukemic evolution. In the era of epigenetics, the possibilities offered by the new molecular targeted therapies offer the chance of modifying the natural history of this disease. Among these, the hypomethylating agents Azacitidine and Decitabine have been shown to induce responses in CMML patients. Azacytidine is mostly incorporated into RNA and reaches DNA following reduction by ribonucleotide reductase, while decitabine is directly incorporated into DNA. Both drugs have been shown to produce a direct decrease of DNA methyltransferase activity in vitro and in vivo, reverting aberrant DNA methylation and increasing the expression of silenced genes, leading to cellular differentiation and/or apoptosis.^3^–^4^ Furthermore a direct cytotoxic effect has been shown in cell lines treated with decitabine, partially explaining the lack of correlation between degree of demethylation and response.^3^–^4^

## Case Report:

We treated 3 CMML-2 patients (2 men and 1 woman) of a median age of 63 years (range 54–69), and IPSS intermediate-2/high (normal caryotype, 1 or two cytopenias and 9–15% bone marrow blasts)(Table 1). Patients were treated with 5-azacitidine (Vidaza, Celgene™) at 75 mg/mq for 7 days at 14.2, 21.6 and 4 months from initial diagnosis. After 4 cycles of azacitidine, 2 patients achieved partial response and are in continuous partial response, with stable peripheral blood counts (Table 1) at 29 and 30 cycles from treatment start and at 36 and 50 months from disease onset. Treatment has been well tolerated, with only mild gastrointestinal and cutaneous toxicity (WHO grade 1). One 54 year-old patient, after a transient response to 5-Azacitidine with normalization of peripheral blood counts, progressed to AML after 4 cycles, underwent allogenic bone marrow transplantation and died for systemic infection at 12 months from CMML diagnosis. During the first azacitidine cycle, this patient had developed a long period of pancytopenia, complicated by acute respiratory distress due to Aspergillus pneumonia, which recovered following anti-fungal treatment. AML progression was characterized by hyperleukocytosis and acquisition of cytogenetic abnormalities. In this patient, younger age, low hemoglobin levels and high peripheral blood monocyte counts at CMML onset, were unfavourable prognostic factors, according to CMML stratified risk assessment.^6^

## Discussion:

Efficacy of Azacitidine and Decitabine in the treatment of MDS has been confirmed by several national and international clinical trials although a protocol comparing efficacy of the two drugs has not been performed yet. These studies demonstrated efficacy of hypomethylating agents compared to best supportive care.^7^–^8^ Azacitidine in particular, significantly prolonged median time to progression to acute myeloid leukemia or death and prolonged median overall survival compared with conventional care regimens.^9^–^12^ Hypomethylating agents have been only rarely used in CMML and there are no prospective studies with sufficient patient numbers in this disease.

A study by the MD Anderson reported the use of decitabine in 19 elderly CMML patients, with encouraging results, with achievement of 58% complete remission (CR) and 11% hematological improvement (HI), resulting into an overall response rate of 69%.^13^ A retrospective review analyzing four phase 3 and phase 2 trials in CMML patients demonstrated 25% overall response rate (CR + PR), with additional 11% HI and 39% stable disease, confirming the superiority of decitabine over best supportive care. Tolerability was good, non hematologic side effects were minimal and myelosuppression-associated complications were acceptable.^14^ A recent paper on 38 CMML treated with Azacitidine at 75 mg/mq/day for 7 days or 100 mg/mq/day for 5 days every 4 weeks, reported 39% overall response rate, with 11% CR, 3% PR and 25% HI resulting into a median overall survival of 12 months.^15^ The median number of cycles given to achieve response was 2, with a median short duration of response of 6.5 months (range, 3 to over 50 months). Incorporation of azacitidine into DNA makes its effect S-phase dependent and requires two or more cycles of DNA synthesis to alter gene transcription and expression. Azacitidine also acts as a biologic response modifier exerting a cytotoxic effect on regulatory T cells or other modulatory cells which may inhibit hematopoiesis in MDS. Thus, the response is subjective and three to four treatment cycles are necessary before the effect becomes clinically apparent. Moreover, the issue of the best treatment schedule is still controversial. Costa et al, tested a dose of 100 mg/mq/day for 5 consecutive days considering this approach more convenient and equal in efficacy.^15^ Todate there are no specific indications on the best treatment schedule in CMML. Due to reversibility of hypomethylating agents effects and to the lack of eradication of the malignant clone, treatment should be continued until response persists.^16^

Our findings confirm highly favorable response rates to azacitidine therapy in high-risk CMML patients, encouraging the application of the treatment in this subset of patients. A correct risk assessment requires the identification of new molecular and clinical features predictive of reponse to hypomethylating agents, to make an effective patient-targeted approach feasible.

## Figures and Tables

**Table 1:** Patients’ Characteristics

	**Patient 1**	**Patient 2**	**Patient 3**

Counts at diagnosis			
Hb (g/dl)	9.9	12	8
WBC (10^9^/L)	19.4	10.5	9.4
Monocytes (10^9^/L)	6.5	3	4
PLTS (10^9^/L)	55	77	178
BM-blasts (%)	11	15	9

Counts after 4 AZA cycles			
Hb (g/dl)	13.3	11.2	Progression
WBC (10^9^/L)	7.09	6.03	
Monocytes (10^9^/L)	0.68	1.03	
PLTS (10^9^/L)	86	84	
BM-blasts (%)	2.5	3.5	

Counts after 8 AZA cycles			
Hb (g/dl)	12.9	11.2	Progression
WBC (10^9^/L)	5.72	4.09	
Monocytes (10^9^/L)	0.70	1.5	
PLTS (10^9^/L)	74	56	
BM-blasts (%)	5	2	

AZA: 5-azacytidine (Vidaza, Celgene™), Hb: hemoglobin; WBC: white blood cells; PLTS: platelets; BM-Blast: bone marrow blast counts
